# Absent or impaired rectoanal inhibitory reflex as a diagnostic factor for high-grade (grade III–V) rectal prolapse: a retrospective study

**DOI:** 10.1186/s12876-021-01729-1

**Published:** 2021-04-07

**Authors:** Byung-Soo Park, Sung Hwan Cho, Gyung Mo Son, Hyun Sung Kim, Yong-Hoon Cho, Dae Gon Ryu, Su Jin Kim, Su Bum Park, Cheol Woong Choi, Hyung Wook Kim, Tae Un Kim, Dong Soo Suh, Myunghee Yoon, Hong Jae Jo

**Affiliations:** 1 Department of Surgery, Pusan National University Yangsan Hospital, Yangsan, Republic of Korea; 2 Department of Internal Medicine, Pusan National University Yangsan Hospital, Yangsan, Republic of Korea; 3 Department of Radiology, Pusan National University Yangsan Hospital, Yangsan, Republic of Korea; 4 Department of Obstetrics and Gynecology, Pusan National University Hospital, Busan, Republic of Korea; 5 Department of Surgery, Pusan National University Hospital, 179 Gudeok-ro, Seo-gu, Busan, 49241 Republic of Korea

**Keywords:** Rectal prolapse, Rectoanal inhibitory reflex, Diagnosis, Manometry, Defecography

## Abstract

**Background:**

Clinically diagnosing high-grade (III–V) rectal prolapse might be difficult, and the prolapse can often be overlooked. Even though defecography is the significant diagnostic tool for rectal prolapse, it is noticed that rectoanal inhibitory reflex (RAIR) can be associated with rectal prolapse. This study investigated whether RAIR can be used as a diagnostic factor for rectal prolapse.

**Methods:**

In this retrospective study, we evaluated 107 patients who underwent both anorectal manometry and defecography between July 2012 and December 2019. Rectal prolapse was classified in accordance with the Oxford Rectal Prolapse Grading System. Patients in the high-grade (III–V) rectal prolapse (high-grade group, n = 30), and patients with no rectal prolapse or low-grade (I, II) rectal prolapse (low-grade group, n = 77) were analyzed. Clinical variables, including symptoms such as fecal incontinence, feeling of prolapse, and history were collected. Symptoms were assessed using yes/no surveys answered by the patients. The manometric results were also evaluated.

**Results:**

Frequencies of fecal incontinence (*p* = 0.002) and feeling of prolapse (*p* < 0.001) were significantly higher in the high-grade group. The maximum resting (77.5 vs. 96 mmHg, *p* = 0.011) and squeezing (128.7 vs. 165 mmHg, *p* = 0.010) anal pressures were significantly lower in the high-grade group. The frequency of absent or impaired RAIR was significantly higher in the high-grade group (19 cases, 63% vs. 20 cases, 26%, *p* < 0.001). In a multivariate analysis, the feeling of prolapse (odds ratio [OR], 23.88; 95% confidence interval [CI], 4.43–128.78; *p* < 0.001) and absent or impaired RAIR (OR, 5.36; 95% CI, 1.91–15.04, *p* = 0.001) were independent factors of high-grade (III–V) rectal prolapse. In addition, the percentage of the absent or impaired RAIR significantly increased with grading increase of rectal prolapse (*p* < 0.001). The sensitivity of absent or impaired RAIR as a predictor of high-grade prolapse was 63.3% and specificity 74.0%.

**Conclusions:**

Absent or impaired RAIR was a meaningful diagnostic factor of high-grade (III–V) rectal prolapse. Furthermore, the absent or impaired reflex had a positive linear trend according to the increase of rectal prolapse grading.

## Background

Rectal prolapse is a disease in which a part of the rectum or the entire rectum slides out through the anus [1]. Rectal prolapse consists of external rectal prolapse (ERP) and internal rectal prolapse (IRP) [2]. Rectal prolapse is diagnosed when the rectum has protruded through the anal sphincter as observed by physical examination. Another way of diagnosing rectal prolapse, especially IRP, is by radiologic defecography. Recently defecography is commonly used as a diagnostic tool for rectal prolapse grading. ERP and high-grade (III–IV) IRP are indications for surgical treatment once the conservative treatment fails [2].

However, patients with rectal prolapse, particularly IRP, sometimes visit outpatient clinics with symptoms such as fecal incontinence or chronic constipation without feeling the prolapse [3, 4]. If a patient does not present with a feeling of prolapse, it is difficult to suspect rectal prolapse, because the symptoms are vague. A physical examination inducing rectal protrusion does not usually work in this situation. Therefore, the clinical diagnosis of rectal prolapse is difficult, and the prolapse can be often overlooked [5, 6]. In addition, defecography, one of the radiologic tests, is considered an unpleasant test for patients [7]. Therefore, it would be clinically useful to have other diagnostic tools or clinical variables to detect rectal prolapse. Among the manometry tests, there are reports that rectoanal inhibitory reflex (RAIR) is associated with rectal prolapse [8, 9]. If RAIR can be a diagnostic factor for rectal prolapse, it may have important clinical significance.

Therefore, we aimed to identify the clinical factors that could detect rectal prolapse, and to investigate whether RAIR can be used as a diagnostic factor for rectal prolapse.

## Methods

### Patient population

Patients who underwent both anorectal manometry and defecography for fecal incontinence, constipation, or anorectal discomforts at Pusan National University Yangsan Hospital between July 2012 and December 2019 were included. All data were taken from a prospectively maintained database. Patients younger than 18 years were excluded. The study design was approved by the Institutional Review Board of the Pusan National University Yangsan Hospital (No. 05-2020-106) and was conducted in accordance with the Declaration of Helsinki.

**Fig. 1:**
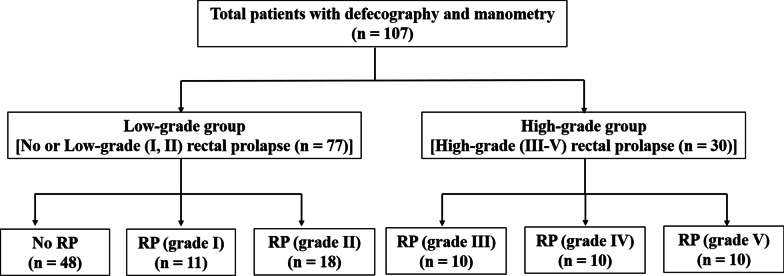
Patient grouping. RP, rectal prolapse

In this study, rectal prolapse was classified in accordance with the Oxford Rectal Prolapse Grading System [4], which defines high-grade (III–V) rectal prolapse as clinically significant rectal prolapse because it requires surgical treatment when it affects quality of life or does not improve with conservative treatment [2]. Thus, patients with high-grade (III–V) rectal prolapse were designated for the high-grade group (n = 30), and patients without rectal prolapse and those with low-grade (I, II) rectal prolapse were designated for the low-grade group (n = 77) (Fig. 1). The Oxford Rectal Prolapse Grading System details are delineated with the description of defecography later in this article.

### Clinical data selection

Clinical variables, including age, sex, symptoms at presentation, and surgical or radiotherapy history were collected. Symptoms at presentation included fecal incontinence, incomplete evacuation, straining, feeling of prolapse, and anorectal pain, which are thought to be associated with rectal prolapse [4]. Symptoms were evaluated using yes/no surveys, answered by the patients. According to Rome IV criteria [10] fecal incontinence was defined as recurrent uncontrolled passage of liquid or solid stool; anorectal pain included levator ani syndrome, unspecified functional anorectal pain, and proctalgia fugax. Incomplete evacuation, straining at defecation and feeling of prolapse were adopted as symptoms related to rectal prolapse [4]. Surgical history included anal surgery, rectal surgery, and hysterectomy.

### Anorectal manometry

Manometry was performed in the left lateral decubitus position. A flexible water-perfused eight-channel catheter with an external diameter of 5.5 mm was inserted into the rectum up to 6 cm from the anal verge. The catheter was connected to a computerized gastrointestinal tract motility recording system (Polygraf ID^®^, Sierra Scientific Instruments, Los Angeles, CA, USA) and the microcapillary infusion system (MUI Scientific, Mississauga, ON, Canada). The capillaries were perfused with distilled water at a rate of 0.5 ml/min per channel using a constant pressure of 96 kPa. A continuous pull-through technique was performed at a rate of 1 cm/s. All patients had an enema 1 h before the testing to prevent fecal content in the rectum from impairing adequate positioning of the catheter.

Maximum resting pressure, mean resting pressure, maximum squeezing pressure and high-pressure zone (HPZ) were evaluated. The maximum resting pressure was defined as the highest resting pressure recorded [11]. The mean resting pressure was considered as the mean of the resting pressures recorded within the HPZ. Moreover, the maximum squeezing pressure was defined as the highest pressure recorded of the anal canal during a maximum squeezing effort by the patient. HPZ was defined as the anal area with a pressure exceeding 50% of the average maximum pressure measured [12].

To measure rectal sensitivity, the catheter with a latex balloon in the tip was inserted into the rectum. Then, the catheter balloon was insufflated with an increment of 10 ml. The first-sensation volume was defined as the volume at which balloon expansion was first felt, and the urge-sensation volume was defined as the volume at which the patient felt a desire to defecate [11, 12]. The maximal tolerated volume was defined as the volume at which the patient felt discomfort and an intense urge to defecate.

**Fig. 2:**
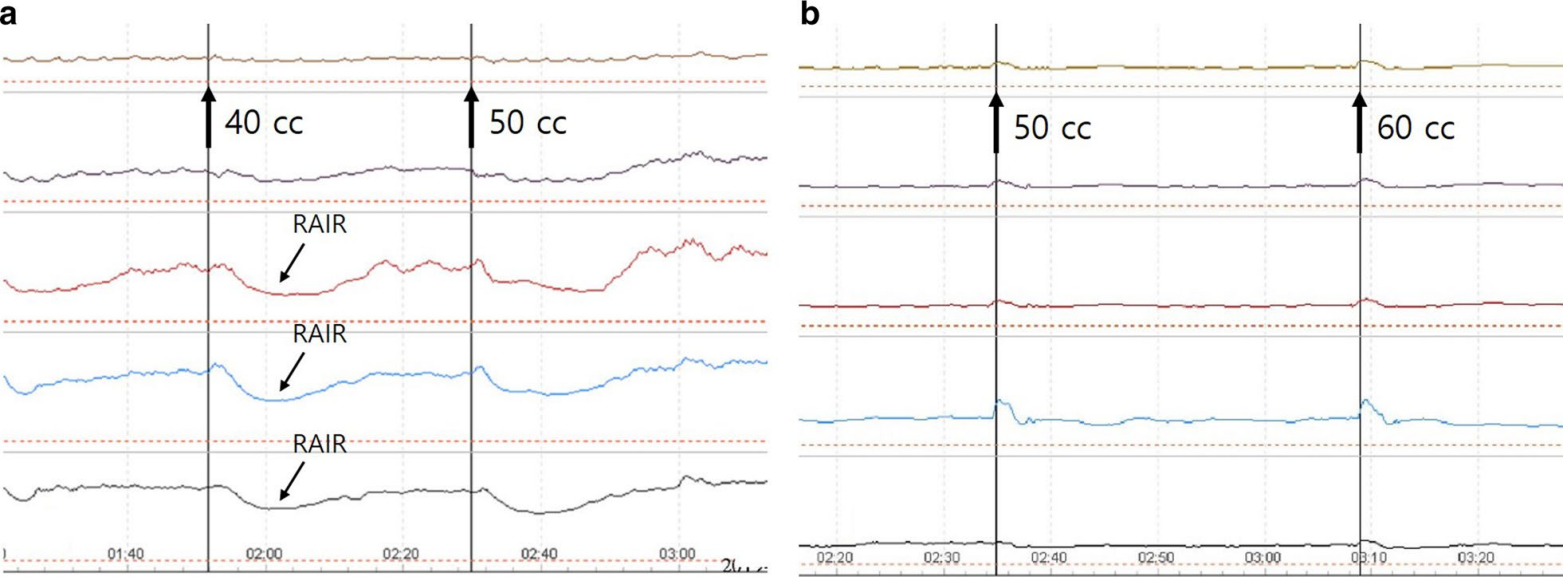
Example of rectoanal inhibitory reflex (RAIR). **a** Normal RAIR. This shows anal relaxation responding to rectal balloon distension. **b** Absent or impaired RAIR. This shows no anal relaxation despite rectal balloon distension

The RAIR was assessed by checking the relaxation of the internal anal sphincter by rapid inflation of the balloon at the catheter tip in the distal rectum. The eight-channel catheter with a latex balloon was reinserted into the anal canal and the balloon was positioned in the distal rectum. After attaining steady pressure, the balloon was rapidly inflated using air with incremental steps of 10 ml, starting from 10 to 60 ml. If the reflex was not elicited with 60 ml, the catheter was repositioned and the procedure was repeated in 60 ml. RAIR was considered to be normally present when the amplitude of resting anal pressure was reduced by 25% or more in response to the rapid inflation of the rectal balloon (Fig. 2a) [11]. Cases in which RAIR was not present or less elicited, even after inflating the balloon to 60 ml were considered to be absent or impaired RAIR, respectively (Fig. 2b). Normal RAIR and abnormality of RAIR were automatically reported by the software installed in the anorectal manometry system. The results were finalized by one gastroenterologist and one colorectal surgeon after blinded verification without noticing any other results including defecography.

The vector volume and asymmetry of the anal sphincter were evaluated by withdrawing the catheter gradually at a rate of 1 cm/s. The two parameters were automatically calculated using the software installed in the computerized anorectal manometry system. Due to the parameters being very sensitive to the catheter position, the anal sphincter pressure was evaluated three times before the catheter position was decided. The asymmetry of the anal sphincter was defined as the degree of deviation of the integrated cross-section from a perfect circle [13]. It was calculated at the HPZ and presented as a percentage (0%, perfect symmetry; 100%, total asymmetry). A higher percentage meant a greater degree of asymmetry. The vector volume of the whole anal sphincter was defined in terms of a 3D shape generated a volume of the overall anal canal [12].

### Defecography

In the fluoroscopy room, the patient’s rectum was filled with 200–250 ml of barium sulfate suspension (70%) introduced by a soft diagnostic enema probe. The patient was then seated on a radiolucent commode placed on the fluoroscopic X-ray table. Lateral video radiographs were taken and recorded at rest, during squeezing, and expulsion of barium enema.

**Fig. 3:**
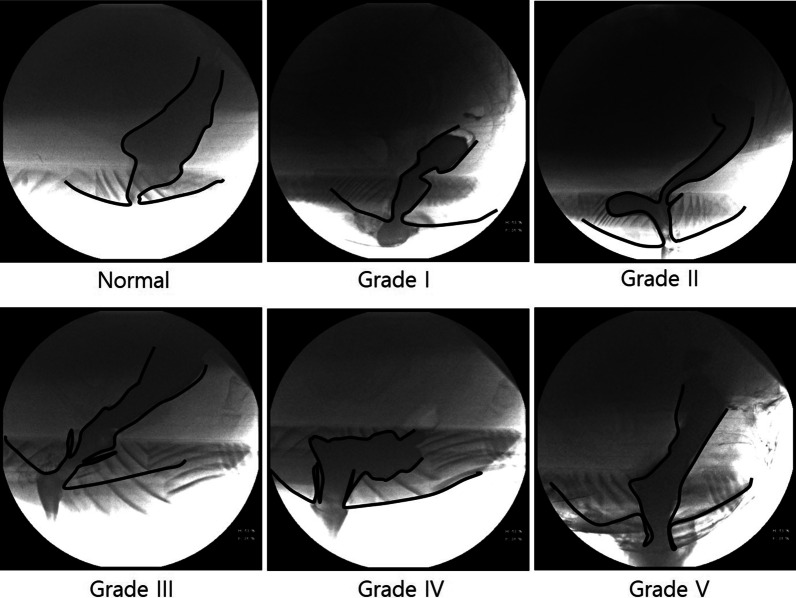
The Oxford Rectal Prolapse Grading System on defecography

The stored defecography videos were analyzed to confirm the presence of rectal prolapse. Rectal prolapse was graded according to the Oxford Rectal Prolapse Grading System (Fig. 3) [4]: grade I, descends no lower than the upper level of a concurrent rectocele; grade II, descends lower than the upper level of a rectocele but not onto the anal canal; grade III, descends and impinges on the anal canal; grade IV, enters the anal canal; grade V, the rectal wall protrudes from the anus beyond the anal verge.

Grade I and grade II prolapse were considered as low-grade IRP, grade III and IV prolapse as high-grade IRP, and grade V prolapse as ERP.

The results were verified by one radiologist and one colorectal surgeon after being blinded without noticing any other results including anorectal manometry.

### Statistical analyses

Statistical analyses were performed using the IBM SPSS Statistics for Windows, version 26.0 (IBM Corp., Armonk, NY, USA). The means of continuous variables were compared using independent t-tests, and non-normally distributed data were analyzed with the Mann–Whitney test. A normal distribution was assessed using the Shapiro–Wilk test. The associations of categorical variables were analyzed with the chi-square test and Fisher’s exact test. Factors that were significant in the univariate analysis were entered in the multivariate logistic regression using a forward likelihood ratio approach. The Cochran-Armitage test for trend was used to check if the change in percentage of the dependent variable had a significant trend according to the changes in the independent variables. Results with *p* < 0.05 were deemed significant.

## Results

### Comparison of clinical characteristics according to the rectal prolapse grading

The percentage of patients with fecal incontinence (18 cases, 60% vs. 21 cases, 27%, *p* = 0.002) and feeling of prolapse (11 cases, 37% vs. 2 cases, 3%, *p* < 0.001) was significantly higher in the high-grade group than in the low-grade group (Table 1). In addition, the proportion of patients with a history of hysterectomy was higher in the high-grade group (*p* = 0.028). There were no significant differences in age, sex, and symptoms such as incomplete evacuation, straining, and anorectal pain. Moreover, there were no significant differences in the history of anal surgery, rectal surgery, and radiotherapy.

**Table 1:** Clinical characteristics of all patients

Parameters	High-grade group (n = 30)	Low-grade group (n = 77)	*p* value
Age (years)	64.8 ± 15.4	61.5 ± 14.5	0.303
Sex (male/female)	10/20 (33/67)	34/43 (44/56)	0.307
Symptoms at presentation			
Fecal incontinence	18 (60)	21 (27)	0.002
Incomplete evacuation	17 (57)	48 (62)	0.589
Straining	17 (57)	49 (64)	0.505
Feeling of prolapse	11 (37)	2 (3)	< 0.001
Anorectal pain	7 (23)	17 (22)	0.889
Past anal surgery (+)	6 (20)	21 (28)	0.417
Past rectal surgery (+)	3 (10)	2 (3)	0.133
Past hysterectomy (+)	6 (20)	4 (5)	0.028
Previous radiotherapy (+)	2 (7)	0 (0)	0.077

Data are presented as mean ± standard deviation or numbers with percentages in parentheses unless otherwise indicated. High-grade group includes grades III–V rectal prolapse. Low-grade group includes no grade and grades I–II rectal prolapse

### Analysis of manometric results between the high-grade group and the low-grade group

The maximum resting pressure, mean resting pressure and maximum squeezing pressure were significantly lower in the high-grade group than in the low-grade group (*p* = 0.011, *p* = 0.009, and *p* = 0.010, respectively) (Table 2). The percentage of patients with absent or impaired RAIR was significantly higher in the high-grade group than in the low-grade group (19 cases, 63% vs. 20 cases, 26%, *p* < 0.001). The vector volumes of the total anal sphincter at rest and squeeze were significantly lower in the high-grade group (*p* = 0.002 and *p* = 0.006, respectively), whereas there were no significant differences in length of HPZ, rectal sensation volume, and asymmetry of the anal sphincter.

**Table 2:** Manometric results between the high-grade group and the low-grade group

Parameters	High-grade group (n = 30)	Low-grade group (n = 77)	*p* value
Maximum resting pressure (mmHg)	77.5 ± 27.3	96.0 ± 35.1	0.011
Mean resting pressure (mmHg)	47.3 ± 20.0	60.9 ± 24.9	0.009
Maximum squeezing pressure (mmHg)	128.7 ± 62.8	165.0 ± 64.4	0.010
Length of HPZ (cm)	2.7 ± 0.8	2.7 ± 0.8	0.731
Rectal sensation volume			
First sensation volume (ml)	44.3 ± 23.5	50.8 ± 30.1	0.291
Urge sensation volume (ml)	78.7 ± 32.2	86.1 ± 39.3	0.363
Maximal tolerated volume (ml)	120 ± 55.4	132.4 ± 52.2	0.292
*Rectoanal inhibitory reflex*			
Normal	11 (36)	57 (74)	< 0.001
Absent or impaired	19 (63)	20 (26)	
Anal asymmetry of HPZ at rest (%)	19.6 ± 5.1	18.5 ± 5.6	0.349
Anal asymmetry of HPZ at squeeze (%)	16.9 ± 5.4	16.7 ± 6.5	0.907
Anal vector volume at rest, median (IQR) (mmHg^2^cm)	18,575 (10,147–25,145)	30,398 (14,300–54,433)	0.002
Anal vector volume at squeeze, median (IQR) (mmHg^2^cm)	48,672 (21,793–83,069)	102,216 (44,449–162,548)	0.006

Data are presented as mean ± standard deviation or numbers with percentages in parentheses unless otherwise indicated. High-grade group includes grades III–V rectal prolapse. Low-grade group includes no grade and grades I–II rectal prolapse. *HPZ* high-pressure zone, *IQR* interquartile range

### Multivariate analysis of the relative factors for high-grade (III–V) rectal prolapse

A multivariate analysis identified feeling of prolapse (odds ratio [OR], 23.88; 95% confidence interval [CI], 4.43–128.78; *p* < 0.001) and absent or impaired RAIR (OR, 5.36; 95% CI, 1.91–15.04; *p* = 0.001) as independent factors of high-grade (III–V) rectal prolapse (Table 3).

**Table 3:** Logistic regression analysis of the predictive factors for presence of high-grade (III–V) rectal prolapse

Predictor	Univariate analysis			Multivariate analysis		
	OR	95% CI	*p* value	OR	95% CI	*p* value
Feeling of prolapse	21.71	4.43–106.30	< 0.001	23.88	4.43–128.78	< 0.001
Fecal incontinence	4.00	1.65–9.70	0.002			
Past hysterectomy	4.56	1.19–17.55	0.027			
Maximum resting pressure (mmHg)	0.98	0.97–0.99	0.015			
Mean resting pressure (mmHg)	0.97	0.95–0.99	0.012			
Maximum squeezing pressure (mmHg)	0.99	0.98–0.99	0.012			
Absent or impaired RAIR	4.92	2.00–12.11	0.001	5.36	1.91–15.04	0.001
Anal vector volume at rest (mmHg^2^cm)	1.00					
Anal vector volume at squeeze (mmHg^2^cm)	1.00					

*OR* odds ratio, *CI* confidence interval, *RAIR* rectoanal inhibitory reflex

### A trend of absent or impaired RAIR according to the changes in rectal prolapse grading

**Fig. 4:**
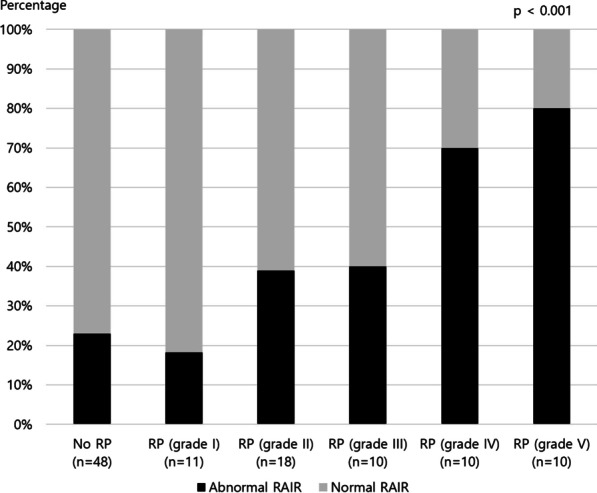
A trend between absent or impaired rectoanal inhibitory reflex and increasing rectal prolapse grade. *RP* rectal prolapse

RAIR was absent or impaired in 23% (11/48) of patients without rectal prolapse and 18% (2/11) of patients with grade I, 39% (7/18) of patients with grade II, 40% (4/10) of patients with grade III, 70% (7/10) of patients with grade IV, and 80% (8/10) of patients with grade V rectal prolapse (Fig. 4). The Cochran-Armitage test for trend confirmed that the percentage of the absent or impaired RAIR significantly increased with increasing grading of rectal prolapse (*p* < 0.001). A sensitivity of absent or impaired RAIR as a predictor of high-grade prolapse was 63.3% and specificity 74.0%.

## Discussion

Our study showed we could use absent or impaired RAIR as a meaningful diagnostic factor of clinically significant high-grade (III–V) rectal prolapse. Furthermore, absent or impaired RAIR had a significant linear tendency to increase rectal prolapse grade. These results may help recognise high-grade rectal prolapse that can be overlooked. RAIR can contribute to enhancing quality of life for patients with the rectal prolapse. Another predictor identified in the present study was the feeling of prolapse.

Our primary focus was whether RAIR was strongly related to rectal prolapse. In the present study, absent or impaired RAIR was the useful diagnostic factor of the high-grade (III–V) rectal prolapse. There have been several studies which showed an association between RAIR and rectal prolapse similar to our study. Spencer reported that all 12 patients with rectal prolapse had absent or impaired RAIR [8]. Similarly, Farouk et al. performed a study of 22 patients with ERP and fecal incontinence [9]. They showed that 16 (73%) patients did not elicit RAIR. In contrast, several reports had results different from those of our study. A study on 27 patients with rectal prolapse reported that 70% of the patients had normal RAIR [14]. Sainio et al. [15] reported that patients with ERP could elicit normal RAIR in 79% (22/28), which was higher than that of our study.

As shown above, the relationship between RAIR and rectal prolapse remains unexplained. Most previous studies limited the study population to patients with ERP, and did not include IRP. Furthermore, most studies had been published before the introduction of the Oxford Rectal Prolapse Grading System which is currently widely utilized in clinical settings [2]. This study classified the patients according to the existence of high-grade (III–V) rectal prolapse for which surgical treatment can be helpful. Therefore, the results could increase the clinical application. Also, this study performed a multivariate logistic regression analysis, which could minimize the influence of confounding variables such as sphincter tone. Finally, it can raise the value on the identified predictors. The linear trend of a positive linear correlation between RAIR and increasing rectal prolapse grading is also a new finding. To the best of our knowledge, this result has never been published to date. This suggests that RAIR might be impaired due to accumulated impact as rectal prolapse progresses.

There are several hypotheses regarding the mechanism behind absent or impaired RAIR in patients with rectal prolapse. One of the suggested mechanisms is that a prolapsed rectum causes persistent RAIR by substantial rectal distention produced by a prolapsed rectum [16]. Therefore, the internal anal sphincter might be relaxed constantly, and RAIR might not be elicited. Many authors have agreed to this hypothesis [9, 17–19]. The hypothesis must be feasible in the case of ERP and was supported by an ambulatory assessment of combined electromyography and anorectal manometry [20]. However, there seems to be a little difference in the case of IRP. In high-grade (III, IV) IRP included in this study, the prolapse usually occurred during the pushing state of defecography. Defecography often shows no specific changes at resting and squeezing state than usual. Therefore, we cannot deduce that a prolapsed rectum elicits constant RAIR without straining in patients with IRP and that it makes absent or impaired RAIR. Furthermore, in the left lateral position for manometry, prolapse or pelvic descent usually does not occur even in ERP. Therefore, the hypothesis might be not definite and may need to be verified by well-designed studies.

Another possible mechanism is direct damage to the internal anal sphincter by chronic stretching of the pelvic floor from rectal prolapse [14, 21, 22]. Dvorkin et al. [23] found that anal sphincter distortion significantly occurred frequently in the rectal prolapse. They concluded that it may be a response to mechanical stress from rectal prolapse although the course was unknown. Woods et al. [24] showed that anatomical defects in both internal and external anal sphincters are common in patients with ERP. Also, the dysfunctional sphincter might be caused by increased collagen fibril in the anal sphincter probably because of continuous stimulation [23, 25]. This study showed both of the resting anal pressure and the squeezing anal pressure decreased in high-grade rectal prolapse. This indicated that the functions of both anal sphincters decreased in rectal prolapse. The results supported that RAIR might not be elicited because of a dysfunctional internal anal sphincter.

The stretching effect from rectal descent can possibly damage the nervous system of the pelvic floor, including the enteric nervous plexus [26]. Park et al. [27] founded histologic evidence of denervation in the anal sphincter in patients with rectal prolapse. They suggested that the denervation could be due to entrapment or stretching injury of the nerve as a result of rectal descent. A study exploring the mechanism of RAIR showed that nitric oxide appeared to be a neurotransmitter to mediate RAIR and the nerve fiber within the internal anal sphincter contained nitric oxide synthase [28]. Therefore, if the nerve fiber is damaged by rectal prolapse, nitric oxide insufficiency may also affect RAIR. In summary, the mechanisms discussed above might influence the reflex simultaneously.

Several factors can influence the occurrence of RAIR and confound the results of our study. The well-known influencing factors are megarectum and extremely low anal resting pressure [14]. This study assessed rectal sensation volume to check for the presence of megarectum. The volumes were not different between the two groups. In addition, if a patient had very low anal resting pressure, manometry was not likely to show RAIR. Detecting RAIR might be difficult when base pressure amplitude is very low. Another probable reason is that the internal anal sphincter might be already inhibited fully by the prolapsed rectum. Sainio et al. [15] reported the absence of RAIR in six patients with rectal prolapse, which might be because of very low anal resting pressure. Therefore, we compared the incidence of very low mean resting pressure (< 20 mmHg) between the two groups. We found that only one patient in the high-grade rectal prolapse group had very low resting pressure and confirmed there was no significant difference in the pressure.

Another significant factor for rectal prolapse in the present study was anal sphincter pressure. Our results showed that both decreased resting and squeezing anal pressure were associated with rectal prolapse in the univariate analysis. However, they were not drawn as independent predictive factors in the multivariate analysis. There have been several reports that anal pressure was related to the rectal prolapse. Harmston et al. [29] reported that the maximum resting and squeezing pressure significantly decreased in ERP, and maximum resting pressure significantly decreased in IRP than the squeezing anal pressure would. Moreover, Zbar et al. [30] showed that maximum resting and squeezing pressure was significantly lower in patients with pelvic organ prolapse. Hawkins et al. [1] reported that increasing rectal prolapse grade was related to an increase in the proportion of patients with decreased sphincter pressures. In contrast, there was a report stating that the maximum resting and squeezing pressure were not associated with the IRP grade [31]. The univariate analysis in our study identified the variable with significant relevance and they may be associated with each other. The relationship may be caused by the common point with structural abnormalities. However, we cannot determine definitely whether decreased anal sphincter tone was the etiology of the rectal prolapse or the consequence of prolonged rectal prolapse [24, 32]. The characteristics of rectal prolapse can be different depending upon the mechanisms which cause the prolapse [33], and future studies must analyze these issues.

Several efforts have been devoted to predicting the existence of rectal prolapse. Karlbom et al. [3] reported that clinical examination such as digital rectal examination and rectoscopy can help diagnose IRP. Prichard et al. [34] concluded that rectoanal pressure patterns on high-resolution rectoanal manometry might identify rectal prolapse. Another study using high-resolution anorectal manometry showed that an anterior high-pressure area with an excessive perineal descent was associated with IRP [35]. The investigators suggested that it could help diagnose prolapse. Although the study results are encouraging, they had a limitation regarding patients’ position. It is usually difficult to induce prolapse of the rectum in the left lateral or supine position [6, 35]. Furthermore, it is more difficult when a probe is located in the rectum during high-resolution anorectal manometry. Moreover, an empty rectum can make producing prolapse more difficult [3]. Another study by Grande et al. [36] using vectography suggested that in cases of incontinent patients with anal asymmetry > 20%, a defecography may be helpful to diagnose rectal prolapse.

Patients with rectal prolapse present to the hospital because of various symptoms [4]. The patient management strategy is determined after obtaining a patient’s history and performing a physical examination. If the patient has a feeling of prolapse or obstructive defecation, rectal prolapse could be easily suspected and visually confirmed by inducing prolapse. However, if the patient complains of other anorectal symptoms such as fecal incontinence or chronic constipation without a feeling of prolapse, other diseases might be considered. Several guidelines suggested that anorectal manometry can be considered more primarily than defecography in the case of fecal incontinence or chronic constipation [37, 38]. Therefore, anorectal manometry is sometimes performed without defecography based on the symptoms and examination findings at presentation. In such cases, we suggest if manometry shows absent or impaired RAIR, clinicians should consider a possibility of rectal prolapse and perform defecography to confirm rectal prolapse. This diagnostic flow could detect the rectal prolapse earlier and ultimately may enhance the quality of life of patients with rectal prolapse.

Of course, the defecography might reveal no or low-grade rectal prolapse. Therefore, if RAIR is absent or impaired, posibilities other than rectal prolapse should be considered simultaneously. Other conditions that impair RAIR include megarectum, post-proctectomy and colo-anal anastomosis, severely reduced internal anal sphincter pressure, chronic constipation, fecal incontinence, rectal ischemia, systemic sclerosis, diabetic neuropathy, myelomeningocele, and Chagas disease [39–42]. However, the aforementioned diseases such as systemic sclerosis and Chagas disease are very rare. In addition, megarectum, postoperative change, and severely reduced anal sphincter were just results that originated from other problems. Although there are many possible causes of impaired RAIR, suspecting rectal prolapse with absent or impaired RAIR on anorectal manometry can have significant clinical implications because prolapse is not very infrequent and active management such as surgical treatment is of great benefit to the patient.

This study has several limitations. First, this was a retrospective study; hence, there is the possibility of selection bias. Prospective studies may be able to obtain more meaningful results. Second, the sample size was small. Therefore, a type II error may have occurred and a larger sample size may have shown statistical significance. Third, this study was conducted in a tertiary hospital; thus, the patients had higher severity than patients in primary and secondary healthcare facilities, which can be another factor contributing to the selection bias.

## Conclusion

Absent or impaired RAIR was a meaningful diagnostic factor of high-grade (III–V) rectal prolapse. Furthermore, the absent or impaired reflex had a positive linear trend according to the increase of rectal prolapse grading.

## Data Availability

The datasets generated and analyzed during the current study are not publicly available due to health privacy concerns, but are available from the corresponding author on reasonable request.
